# Public Service for Children

**Published:** 1911-06-15

**Authors:** J. P. Root


### PUBLIC SERVICE FOR CHILDREN.

BY J. F. ROOT, D.D.S.

A great deal is being said upon the subject of "Public Service for Children," and comparing the sayings and doings, the latter suffers by comparison. A few cities are in the Doing Department, but most towns, villages and cities are lying dormant as far as the Public Service of Oral Hygiene is concerned. In some localities the establishing of free dental infirmaries is delayed until the question of whether or no amalgam should be used in preference to gold or porcelain.

Dr. O. Ottolengui, editor of the *Items of Interest*, as well as the manufacturer of theories and fads, has set, and peculiar ideas regarding free dental clinics, he says in the January *Items:* "*The point intended to be brought out is that gold and porcelain should be used in the free clinic in exactly the same ratio to amalgam as they are used in private practice. Or in other words, the dentist when serving the poor should follow the example of his brother the physician, and render the same service in the clinic room as he does in his private office.*"

New York is our greatest city and contains more children in need of free dental service than any other, and to date has displayed less enthusiasm in establishing free dental infirmaries than most large cities. The reason for this neglect is very apparent, if other dentists of that city believe with Dr. Ottolengui: that no distinction should be made in the material used in filling the teeth of the poor and rich, he cites the practice of the physician who makes no distinction in his treatment of the poor. In many instances this is true, for the pampered son of Mrs. Fifth Avenue requires the same dose of calomel to carry away the mince pie as does the pauper son of Mrs. Bowery to loosen the staying powers of some hot tamale, yet one is probably administered with a sugar coating and gentle persuasion, the other in shirt sleeves, and with forcible coercion, and the results are the same, but if Mrs. Fifth Avenue has an offending appendix to be plucked out, she wends her way to the hospital in a rubber-tired ambulance; has all of the attention and luxurious surroundings money can pay for, her convalescence, while not a continuous round of pleasure, would be, to the poor woman from the bowery, whose appendix has been taken away and she, passed on to a space in the general ward, to while away her time in convalescing. This is the normal custom, and not a strange one. Both had an appendix, both were removed, and you could not tell them apart.

The operation itself was as carefully performed in one case as the other, but as a comparison one had money and after the cavity was prepared had a gold inlay inserted; the other being poor had just as careful preparation and an amalgam filling, and the results were equally good, but suppose this New York surgeon had ideas similar to some dentists, and believed Mrs. Pauper should have all of the luxuries of Mrs. Fifth Avenue, and refrained from operating until they were provided, who would provide them? and while waiting what would be the result? Dr. Ottolengui says: "'*If amalgam will save a tooth as well as gold, why do you ever use gold?' There is but one reply to this from an ethical practitioner who does not fill teeth for the sake of the fee rather than for the salvation of the organ itself, and that answer is that 'There are places where gold will save teeth better than amalgam.' If this be true in private practice, it is likewise true in the clinic room, and consequently we are not giving the poor 'the acme of professional attention' if gold is barred entirely.*"

If there is a dentist on earth with whom the question of the fee is not one considered, I have never met him or heard of him and doubt if he exists. There are physicians and surgeons who practice simply for the love of humanity, and the difference between the practice of medicine and dentistry is apparent enough to show why dentists do not do likewise.

It would be just as sane an act if charitable institutions refrained from providing clothing for the poor, because they felt that silks and satins should be given, instead of wool or cotton, and not being able to provide the former, allowed nakedness and suffering to exist.

Dr. Ottolengui asks: "*What would we think of a physician who denied his patient the benefit of an expensive drug because the sick man did not have the price of the best remedy?*" The vital necessity of a certain remedy in case of sickness is so apparent, when perhaps a life depends upon its ministration, that to compare it to the loss of a tooth is a childish question and needs no answer, except perhaps to say as you would to the child, "Wait until you grow up, then perhaps you can understand such a proposition."

Dr. Ottolengui is tempted, for he says: "*It may be true that ninety per cent of cavities in molars and bicuspids are filled with amalgam, but it certainly is not true that these teeth are 'saved,' in the sense that they would be saved if properly filled with gold. If the truth could be known, the probabilities are that fully ninety per cent of these amalgam fillings permit recurrence of caries within five years. The writer is strongly tempted to undertake the task of proving this statement if only to persuade the dental world that the easiest way is not always the best way.*"

The above statement is one difficult to prove or disprove, yet it is probably true. The belief that the average life of all fillings is not to exceed five years is a true one, and Dr. Ottolengui will confer an everlasting favor upon the dental profession if he will do as he is tempted; that is, prove that ninety per cent of all amalgam fillings are failures within five years, but he must bear in mind that he must base his report upon facts, not theories, and he must also obtain the assistance of such an income as that of the Standard Oil Company to be able to gather the data. He inserts one saving clause in his statement, "*But it certainly is not true that these teeth are 'saved' in the sense they would be if properly filled with gold.*" Kindly notice the insertion of the word *properly*, and then from past personal observation estimate how many fillings are properly inserted. It is my belief from experience and personal observation that there are ten teeth saved with amalgam to one with gold—when the teeth are bicuspids or molars with approximal cavities. This is easily explained by considering the difficulty of successfully adapting gold at the cervical margin compared to the ease with which amalgam can be used. He says: "*But if expense must be considered, it is not necessarily true that amalgam is either a less painful or a less protracted method than gold, for no amalgam filling, properly inserted, causes less pain in the preparation of the cavity than does the preparation of the same cavity for a gold inlay. and the placing of an inlay is no more protracted than the placing of an amalgam*

*filling plus the polishing of the amalgam, which should be done at a subsequent sitting."*

The belief that the preparation of a vacity for a gold inlay is no more painful than for amalgam is a strange one and not based on facts, for we all know the pain of excavation is in the cutting of sound tooth structure, and in case of an inlay the cutting must be far more extensive than for amalgam, especially in approximal cavities in molars or bicuspids. This statement is absurd and needs no refutting.

He argues that: *"If expense must be considered in the dental clinic room, let it be known that the best and cheapest method of using amalgam is to save the cost of the mercury, purchase the alloy in bulk, and fill the teeth with cast alloy inlays. In this way we will have the benefit of the cement lining to the cavity, and what is more important, we may prepare the cavities with proper extension for prevention and with proper marginal protection, since the alloy is inserted hard, whereas the amalgam is inserted in a plastic condition. Moreover, the edge strength to the cast alloy is greater than can be had with amalgam."*

The use of cast alloy inlays is a new one, and does not seem to fill a long-felt want, for if a cheap material is needed for this purpose, there are several on the market apparently more appropriate than a melted amalgam, and the argument that in such a procedure the result would be a cement lined cavity, is not a good reason, for with the present knowledge of amalgam, no sane man inserts it without first lining the cavity with cement, or inserting it on soft cement.

It seems to me space in dental journals and labor in filling said space could better be devoted to encouraging the establishing of free dental infirmaries, rather than the discouragement, for there are a sufficient number of legitimate obstacles strewing the paths of the promoters of the institutions, the first thing is to establish, then the management and procedures will regulate themselves, mistakes will be made and rectified, but results will come.—*Western Dental Journal.*

